# Perirectal angioleiomyoma preoperatively misdiagnosed as rectal cancer: a case report

**DOI:** 10.3389/fonc.2024.1476084

**Published:** 2024-10-24

**Authors:** Wenhan Liu, Xianxiong Wen, Dongping Hu, Hong Ma

**Affiliations:** ^1^ Department of Anorectal Surgery, Gansu Provincial Hospital, Lanzhou, China; ^2^ Department 1 of Day Diagnosis and Treatment Center, Gansu Provincial Maternity and Child-care Hospital, Lanzhou, China; ^3^ Department of Interventional Medicine, Gansu Provincial Hospital, Lanzhou, China

**Keywords:** angioleiomyoma, case report, disease characteristics, differential diagnosis, rectal cancer

## Abstract

Angioleiomyoma (ALM) is a rare benign perivascular (pericytic) tumor primarily composed of well-differentiated smooth muscle and vascular components. Its clinical and radiological features lack specificity, making diagnosis challenging and prone to misdiagnosis. This report summarizes the clinical data of a patient treated at our hospital who was preoperatively misdiagnosed with rectal cancer but was subsequently found to have perirectal ALM. Additionally, a review of the relevant literature is provided.

## Case report

A 51-year-old female patient was admitted to the hospital, with a chief complaint of a perirectal mass noted for over 10 years. Physical examination (knee-chest position) revealed good contraction of the anal sphincter, no palpable anal canal mass at 5 cm, but a hard, extrarectal mass was palpated at 3 cm from the anal verge. There was no blood or mucus on the glove upon withdrawal. Routine blood tests, liver and kidney function tests, coagulation function tests, infectious disease series, and tumor markers were all normal.

**Figure 1:**
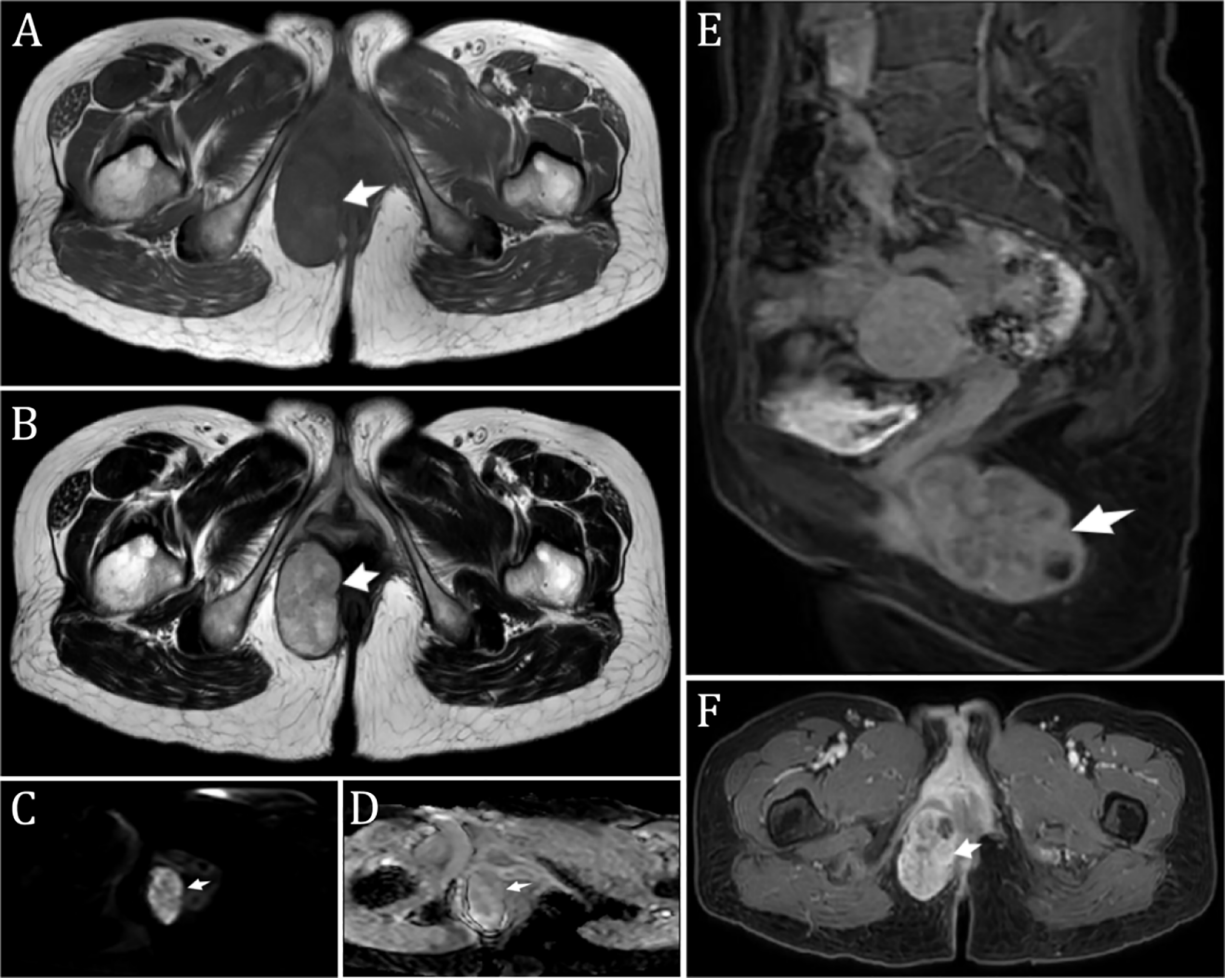
Preoperative Imaging **(A, B)**. T1-weighted image **(A)** and T2-weighted image **(B)** showing a perianal mass in the right gluteal subcutaneous fat. The mass exhibited iso- to slightly hyperintense T1 and T2 signals, with a clear boundary and measuring approximately 5.8 cm × 3.3 cm × 5.0 cm; **(C)** Diffusion-weighted imaging (DWI) displaying high signal intensity; **(D)** Apparent diffusion coefficient (ADC) showing iso-to-low signal intensity; **(E)** Sagittal fat-suppressed contrast-enhanced T1 axial image; **(F)** Enhanced image showing uneven, significant enhancement with patchy areas of liquefactive necrosis within. *The arrow indicates the mass (considering malignancy).

A non-contrast MR scan of the abdomen and pelvis with DWI was performed, showing a mass in the subcutaneous fat of the right buttock. The mass exhibited iso- to slightly hyperintense T1 and T2 signals, high signal on DWI, and iso- to hypointense signal on ADC ((0.95 ± 0.15)×10^-3^ mm^2^/s), with a clear boundary and a size of approximately 5.8 cm × 3.3 cm × 5.0 cm (**Figure 1**). The radiological diagnosis was a right buttock subcutaneous fat mass, and enhanced imaging was recommended.

A n enhanced MR scan of the abdomen and pelvis revealed a mass in the subcutaneous fat of the right buttock with iso- to slightly hyperintense T1 and T2 signals. The enhanced scan showed uneven significant enhancement with patchy liquefactive necrosis within the lesion, measuring approximately 5.8 cm × 3.3 cm × 5.0 cm (**Figure 1**). The diagnostic suggestion was a subcutaneous fat mass in the right buttock, likely a malignant rectal tumor.

Based on preoperative imaging, the preoperative diagnosis was a malignant rectal tumor. The patient underwent radical resection of the malignant rectal tumor under general anesthesia, with intraoperative biopsy of the right perirectal mass for rapid frozen pathology. The intraoperative frozen section revealed a spindle cell tumor of mesenchymal origin. Immunohistochemical staining indicated a high likelihood of a pericytic tumor (suggestive of myopericytoma, with stromal myxoid changes). Immunohistochemical results were: CD117 (-), Dog-1 (-), CD34 (+), S-100 (-), SOX-10 (-), SMA (+), Desmin (+), Vimentin (+), STAT-6 (-), Ki67 (+10%), HMB45 (-), MelanA (-), CK8-18 (-).

**Figure 2:**
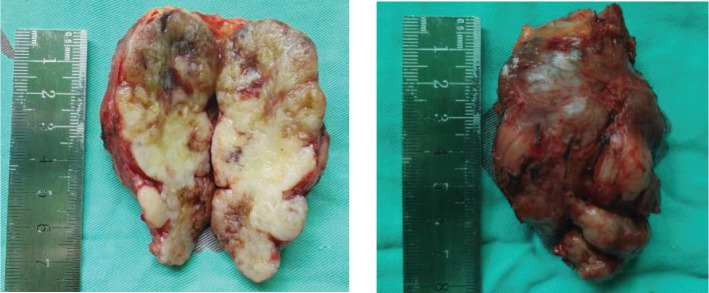
Postoperative Specimen. Gross examination: (Perirectal mass) A gray-brown and gray-white nodular tissue measuring 7.5 x 5.5 x 3.5 cm. The mass was surgically incised and the cut surface was gray-brown and solid in appearance.

**Figure 3:**
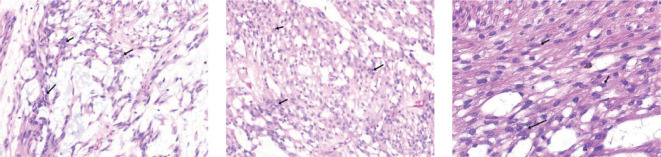
Postoperative Pathology. Immunohistochemistry results: CD34 (scattered +), CD31 (-, positive in blood vessels), Desmin (diffuse +), SMA (diffuse +), S-100 (-), Vimentin (+), CKP (-), Caldesmon (scattered +), TLE-1 (scattered weak +), Rb (-, loss), Muc-4 (-), Catenin-B (-).

The planned surgical procedure was altered, and a simple excision of the perirectal mass was performed successfully (**Figure 2**). Postoperative pathological diagnosis was: (perirectal) mesenchymal tumor, consistent with angioleiomyoma with extensive myxoid changes, based on immunohistochemistry. Immunohistochemical staining results were: CD34 (scattered +), CD31 (-, vascular +), Desmin (diffuse +), SMA (diffuse +), S-100 (-), Vimentin (+), CKP (-), Caldesmon (scattered +), TLE-1 (scattered weak +), Rb (-, loss), Muc-4 (-), Catenin-B (-) (**Figure 3**).

Postoperatively, the patient received symptomatic treatments including fluid replacement, anti-infection, acid suppression, and analgesia. The patient’s condition stabilized, and she was discharged. One month later, a follow-up abdominal ultrasound showed no significant abnormalities. At the two-month follow-up, the patient was in good general condition with no signs of recurrence.

## Discussion

Initially, angioleiomyoma (ALM) was considered a smooth muscle tumor. In 2013, the World Health Organization (WHO) classified it as a perivascular (pericytic) tumor. The peak incidence of ALM is between 40 and 60 years of age. Clinically, the disease often presents as a solitary, slow-growing, firm, occasionally painful cutaneous mass, primarily in the lower limbs, and predominantly occurs in females (1). Literature reports suggest that its size and pain degree may correlate with the menstrual cycle and pregnancy, indicating a potential hormone dependency (2). ALM can occur in various body parts, most commonly in the lower limbs (3), followed by the head and neck region (4), with documented cases in the intracranial and oral regions, there have been two reported cases occurring within the rectum, both of which showed favorable prognoses following simple surgical resection (5, 6), but no reported cases in the perirectal area.

Regarding histogenesis, most scholars believe ALM originates from the tunica media of vein walls, while some consider it a vascular malformation (7) or an intermediate process between hemangioma and solid leiomyoma (4). The pathogenesis of ALM involves two genetic mutation patterns: the first characterized by a t(4;5)(p12;q32) translocation producing the CARMN::TXK fusion gene; the second involves recurrent Xq22 rearrangements leading to IRS4 overexpression (8). Animal studies suggest that N-nitrosoethylurea compounds may induce ALM in the spleen (9).

Clinically, ALM often presents with cold-induced sudden pain, possibly due to ischemia from smooth muscle contraction induced by cold (10). However, this is not a specific symptom; for instance, this case presented no obvious symptoms, only a gradually enlarging, painless mass. Definitive diagnosis relies on postoperative histological examination and immunohistochemistry. Microscopically, ALM typically features numerous dilated blood vessels interspersed with spindle cells and collagen bundles lacking elastic fibers. The smooth muscle fibers around the vascular lumen form a regular ring pattern, and the nuclei of disordered smooth muscle cells appear cigar-shaped with eosinophilic cytoplasm. Mitosis, cytological atypia, necrosis, or pleomorphism are rarely observed. ALM can be pathologically classified into three types (11): (1) Solid/capillary type, the most common, characterized by compact smooth muscle and numerous small, slit-like vascular channels, with a higher incidence in females; (2) Venous type, featuring a thick muscle wall and loosely arranged smooth muscle bundles, more common in males; (3) Cavernous type, the rarest, composed of dilated vascular channels and a few smooth muscle bundles, also more common in males.

Immunohistochemically, ALM typically shows negative staining for epithelial membrane antigen (EMA), serum acidic binding protein S100, glial fibrillary acidic protein (GFAP), and desmin, while smooth muscle actin (SMA) and either CD34 or CD31 are markers for muscle cells and vascular endothelial cells, respectively. These specific immunohistochemical results are crucial for diagnosis and differential diagnosis (12). There have been reports of CD34-negative ALM, speculated to be due to immunohistochemical errors or an unclear CD31(+) status (13). Immunohistochemical examinations conducted in this case included DOG1, which ruled out a diagnosis of GIST, and S-100 protein, which excluded a diagnosis of peripheral nerve sheath tumor.

In imaging, MRI has diagnostic value for ALM. ALM lesions show homogeneous low signal intensity similar to skeletal muscle on T1-weighted images. Solid and venous types exhibit heterogeneous signal intensity on T2-weighted images, appearing isointense or slightly hyperintense, while cavernous types show high signal intensity. Gadolinium-enhanced scans show heterogeneous enhancement (13). Some researchers suggest that a mixed high and isointense signal on T2-weighted MRI with a low signal margin should prompt a diagnosis of angioleiomyoma (14). However, in this case, MRI revealed that the mass exhibited high T1 and T2 signal intensity, which contrasts significantly with previous reports in the literature. This serves as a reminder that during the diagnostic process for such conditions, multiple diagnostic methods, particularly pathology, should be utilized to make a well-informed decision. On ultrasound, ALM typically appears as an oval, well-defined, homogeneous hypoechoic mass. Gray-scale ultrasound shows low echogenicity in the venous type and isoechoic in the solid type. Color Doppler imaging shows rich blood flow in the venous type and relatively poor flow in the solid type, corresponding to their microscopic appearance, thus having value in differentiating ALM subtypes (15). Preoperative CT diagnosis of ALM is extremely challenging, showing irregular, heterogeneous density masses with uneven nodular enhancement (16). Despite the atypical appearance, CT imaging remains indispensable due to its detailed, realistic, and three-dimensional reconstruction capabilities, aiding in precise elective surgical excision and reducing surgical trauma and complications.

For treatment and prognosis, simple excision is the preferred treatment for ALM. Surgical excision alleviates clinical symptoms and, given the high misdiagnosis rate, allows for accurate pathological examination to confirm the diagnosis. There have been cases where angioleiomyoma progressed to angioleiomyosarcoma, indicating that delayed or inappropriate treatment increases the risk of malignancy (17). Therefore, timely and active treatment is necessary. The prognosis is excellent postoperatively, with an extremely low recurrence rate, and no human ALM recurrence cases have been reported. Annual follow-up is recommended after excision (18).

In conclusion, ALM is a rare benign tumor, and its occurrence in the perirectal area is exceedingly rare. Complete excision of the lesion is the preferred treatment for all types, with excellent surgical outcomes. Despite the difficulty in preoperative diagnosis, clinicians must conduct thorough preoperative evaluations and examinations to avoid misdiagnosis and restore patient health with minimal cost.

## Data Availability

The original contributions presented in the study are included in the article/supplementary material. Further inquiries can be directed to the corresponding author.
